# Effectiveness and safety analysis of ketogenic diet therapy for drug-resistant epilepsy caused by structural pathology

**DOI:** 10.3389/fneur.2024.1497969

**Published:** 2024-10-30

**Authors:** Hongwei Zhang, Song Su, Huan Zhang, Lina Sun, Yong Liu, Guohua Liu

**Affiliations:** ^1^Department of Neurology, Children's Hospital Affiliated to Shandong University, Jinan, Shandong, China; ^2^Department of Neurology, Jinan Children’s Hospital, Jinan, Shandong, China; ^3^Epilepsy Center, Children's Hospital Affiliated to Shandong University, Jinan, Shandong, China; ^4^Epilepsy Center, Jinan Children’s Hospital, Jinan, Shandong, China; ^5^Department of Special Function Examination, Anqiu People's Hospital, Weifang, Shandong, China; ^6^Department of Ophthalmology, Children's Hospital Affiliated to Shandong University, Jinan, Shandong, China; ^7^Department of Ophthalmology, Jinan Children’s Hospital, Jinan, Shandong, China

**Keywords:** ketogenic diet, structural etiology, drug resistant epilepsy, effectiveness, safety

## Abstract

**Objective:**

To explore the effectiveness and safety of the ketogenic diet (KD) in children with drug resistant epilepsy (DRE) caused by structural etiology.

**Methods:**

The children were categorized into acquired brain injury group and malformations of cortical development (MCD) group based on the etiology. Follow-up assessments were performed at 1, 3, and 6 months after KD treatment to observe seizure reduction, behavioral and cognitive improvements, adverse reactions events, and reasons for discontinuation withdrawal. Statistical analysis was conducted on the results.

**Results:**

We found the seizure-free rates at 1, 3, and 6 months were 4.8% (2/42), 19% (8/42), and 21.4% (9/42), respectively. The seizure control effective rates were 42.9% (18/42), 52.4% (22/42), and 54.8% (23/42) at the corresponding time points. Compared to the acquired brain injury group, the MCD group showed a higher seizure control effective rate. Further analysis within the MCD group revealed the highest efficacy in focal cortical dysplasia (FCD). At the 3-month follow-up, cognitive and behavioral improvements were observed in 69% (29/42) of children. The main reasons for discontinuation were lack of efficacy and poor compliance.

**Significance:**

Finally, we get that KD is a safe and effective treatment for drug resistant epilepsy caused by structural etiology, with the added benefit of improving behavioral and cognitive abilities in children. The efficacy is higher in children with MCD, particularly in cases of FCD. Early intervention with KD is recommended for this population.

## 1 Introduction

Ketogenic diet therapy (KDT) is a dietary approach with a high proportion of fat, low carbohydrates, and appropriate levels of protein and other nutrients. Currently, four main types of KDT are commonly used: (1) classic KDT, (2) medium-chain triglyceride (MCT) diet or modified MCT diet, (3) modified Atkins diet (MAD), and (4) low glycemic index treatment (LGIT) (1). Its efficacy and safety have been confirmed in various conditions such as autism spectrum disorders, infections, diabetes, and neurometabolic diseases (2) Proposed as a treatment for epilepsy in 1920 (3), KDT has a history of over 100 years and has been used in China since 2004 to treat drug resistant epilepsy (DRE) (4). Extensive research has shown its significant efficacy in DRE, various epilepsy syndromes, and epileptic encephalopathies. While oral anti-seizure medications (ASMs) are the primary treatment for children with epilepsy, some children cannot achieve effective seizure control and develop DRE. Limited data and research exist on the relationship between KDT and epilepsy etiology, especially in children. Structural etiology is a common cause of DRE in children, with acquired brain injury and malformations of cortical development (MCD) being common etiologies, and the acquired brain injury is more common in neonatal hypoxic–ischemic encephalopathy (HIE), hypoglycemia, cerebral hemorrhage, and sequelae of intracranial infection, etc. MCD are a phenotypically and genetically heterogeneous group of disorders, with focal cortical dysplasia (FCD) being the most common type (5–7). For children with DRE caused by structural etiology who are not suitable for or temporarily unwilling to undergo surgery, determining the effectiveness of KDT can help clinicians and patients consider it as an effective method for seizure control. Therefore, this study analyzes the efficacy and safety of KDT in 42 children with DRE related to structural etiology.

## 2 Materials and methods

### 2.1 Study subjects

This is a single-center retrospective study, which examined individuals seen at the hospital from April 2021 to July 2023, selecting patients based on the following inclusion and exclusion criteria. This single-center retrospective study included 42 patients with DRE due to structural etiology who underwent KDT at the Epilepsy Center of Shandong University Affiliated Children’s Hospital from April 2021 to July 2023. All included subjects met the 2010 diagnostic criteria of the International League Against Epilepsy, failure of adequate trials of two tolerated, appropriately chosen and used antiepileptic drug schedules (whether as monotherapies or in combination) to achieve sustained seizure freedom, and had evidence of structural etiology in the brain (8). Informed consent for participation in the study and the ketogenic diet was obtained from each patient or their parents/caregivers. The study was conducted in compliance with the Declaration of Helsinki.

### 2.2 Inclusion criteria

(1) Compliance with the International League Against Epilepsy diagnostic criteria for DRE; (2) Ketogenic diet treatment for more than 3 months; (3) Evidence of structural changes in brain imaging; (4) Ruling out other etiology; (5) Guardian consent for KD and signed informed consent.

### 2.3 Exclusion criteria

(1) Severe diseases affecting the digestive, respiratory, immune, or cardiovascular systems; (2) Presence of contraindications for KD, such as various inherited fatty acid metabolism disorders, and porphyria; (3) Poor treatment compliance.

### 2.4 KD treatment

Prior to KD treatment, a comprehensive medical history and physical examination are conducted for all patients. This involves thorough routine blood, urine, and stool tests, biochemical analyses, blood ammonia and lactate assessments, urine tandem mass spectrometry analysis, as well as electrocardiography, abdominal and Renal ultrasound, video electroencephalography, cranial magnetic resonance imaging, developmental testing evaluations, and other examinations. This aims to rule out contraindications for KD treatment. The classical KD treatment protocol is employed, with a fat-to-(protein plus carbohydrate) ratio of 4:1. Initial energy requirements are determined based on ideal height and weight, typically ranging from 75 to 85%, to maintain optimal seizure control and meet nutritional needs for growth and development. The initiation of KD treatment usually begins with a ratio of 1.5:1–2:1, gradually adjusting to 3:1 to 4:1 based on the child’s age and tolerance. Individualized plans are formulated, maintaining blood ketone levels between 3 and 5 mmol/L, blood glucose between 4 and 5 mmol/L, and urine ketones between 3+ and 4+. Adjustments to dietary proportions and protein intake are made promptly based on monitoring indicators and daily protein requirements. Additionally, supplementation with sugar-free and lactose-free calcium, various trace elements, complex vitamins, and potassium citrate may be necessary.

### 2.5 KD management

After a nutritionist develops an individualized KD menu, caregivers are informed about recording KD effectiveness method and content, including daily monitoring of the child’s blood ketone and blood glucose levels, as well as documenting seizure occurrences, their forms, tolerability, and adverse reactions. Communication with the nutritionist is encouraged during this period for timely adjustments to the treatment diet based on the child’s tolerance and blood ketone and blood glucose levels. Regular outpatient follow-up appointments are scheduled at 1, 3, 6, and 12 months after initiating KD treatment, during which blood tests, biochemical analyses, abdominal ultrasound, and Renal ultrasound are repeated.

### 2.6 Observation indicators

(1) A baseline value of average daily seizure frequency in the 28 days before starting KD is established. Subsequent follow-ups at 1, 3, and 6 months compare the average daily seizure frequency to the baseline, with a reduction of 50% or more considered effective seizure control and 100% reduction indicating seizure freedom. At the 3-month time point, cognitive and behavioral improvements are assessed in comparison to pre-KD initiation. (2) Compliance and safety of KD treatment are assessed by calculating retention rates at 1, 3, and 6 months after KD initiation. The reasons for treatment discontinuation are analyzed, and adverse reactions documented in KD follow-up diaries are assessed. (3) Record the comparison between the average monthly seizure frequency of two groups of pediatric patients before the observation points after enrollment, grouped by structural pathology, and their baseline seizure frequency, to determine the efficacy of seizure reduction. The seizure reduction rate is calculated as follows: [(Baseline average monthly seizure frequency-Average monthly seizure frequency at each observation points after enrollment)/Baseline average monthly seizure frequency] × 100%. The treatment outcomes for epileptic seizures are classified into four grades according to the Engel classification criteria (9): Grade I: Complete control, with no seizures after treatment; Grade II: Significant improvement, with a 90–99% reduction in seizures; Grade III: Improvement, with a 50–89% reduction in seizures; Grade IV: Ineffective, with a reduction in seizures of <50%. The overall effective rate is calculated as: (Number of cases in Engel Grades I, II, and III) /Total number of pediatric patients × 100%. Summarizing the potential prognosis resulting from common seizure frequencies observed in clinical practice, as well as calculating the median of the average daily seizure frequency, we categorize patients into a group with seizure frequency ≥ 5/day. The improvement in cognitive development is comprehensively assessed based on the observations and overall descriptions provided by the parents of the pediatric patients after their inclusion in the study and treatment, as well as conduct developmental assessments using the Gesell Developmental Scales, Peabody Developmental Motor Scales, and Wechsler Intelligence Scales for Children.

Statistical data analysis was conducted using SPSS Statistics 26 software, while statistical results were visualized using Excel and GraphPad Prism 9 software. For measurement data, normality tests were performed using the Shapiro–Wilk test and Kolmogorov–Smirnov test. Normally distributed data were presented as mean ± standard deviation, while skewed distribution data were described using the median and interquartile range M (P25, P75). Count data were expressed in terms of number of cases and percentages. For comparisons of differences between groups for measurement data, the t-test was applied if the data followed a normal distribution, and the rank-sum test was used if the data did not follow a normal distribution. For comparisons of differences between groups for count data, the chi-square test or Fisher’s exact test was applied. A *p* < 0.05 was considered statistically significant.

## 3 Results

### 3.1 Clinical characteristics

This single-center retrospective study included 42 patients with DRE due to structural etiology. Of these, 29 were male and 13 were female, with ages ranging from 2.4 to 129 months and the median age of 20.5 (10.7, 61.0) months. The subjects were classified into acquired brain injury group (nine cases of neonatal hypoxic–ischemic encephalopathy, four cases of neonatal hypoglycemic brain injury, two cases of neonatal cerebral hemorrhage, 1 case of cerebral hemangioma, three cases of sequelae after intracranial infection, for example viral encephalitis etc., two cases after intracranial surgery, for example tumor resection, and two cases cerebral hemorrhage and four other case) and MCD group (nine cases of FCD, three cases of megalencephaly, two cases of tuberous sclerosis and one case of gray matter heterotopia). The study was approved by the Ethics Committee of Shandong University Affiliated Children’s Hospital.

**Figure 1:**
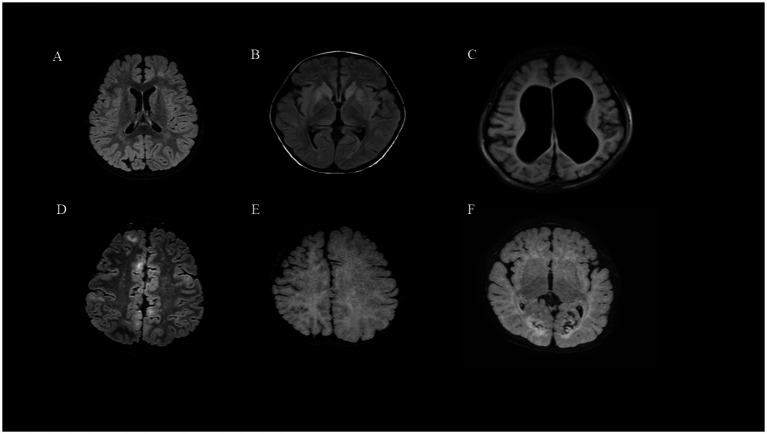
Brain MRI findings of DRE due to structural etiology: (A) (Group2, Patient 2, FCD). (B) (Group1, Patient 15, Intracranial infection). (C) (Group1, Patient 4, HIE). (D) (Group2, Patient 16, tuberous sclerosis). (E) (Group2, Patient 13, lissencephaly). (F) (Group1, Patient 12, Hypoglycemic encephalopathy).

**Figure 2:**
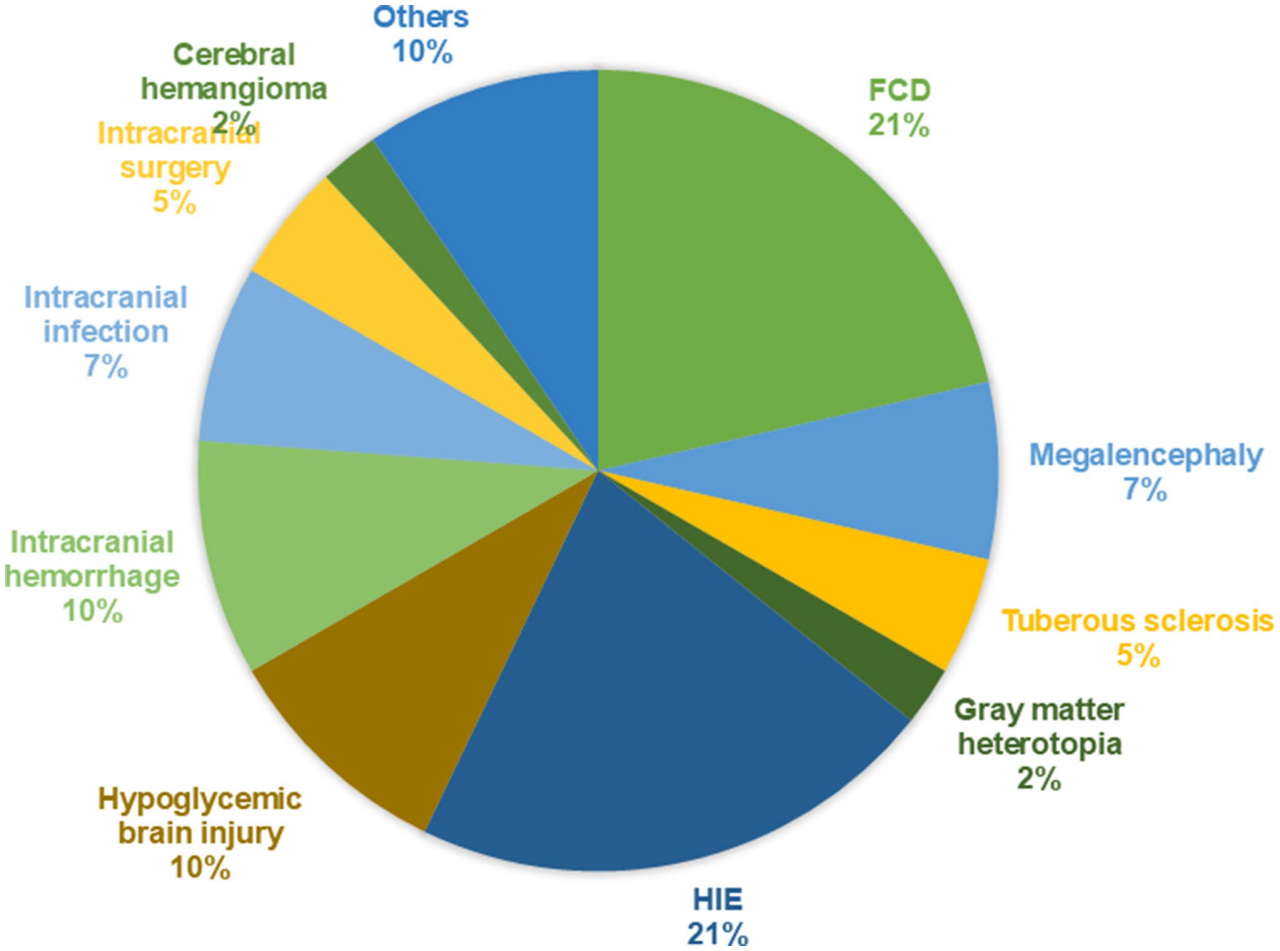
The proportion of patients with each disease among the 42 cases.

Among the 42 patients, there were 29 males (69.0%) and 13 females (31.0%), with ages ranging from 2.4 to 129 months and a median age of 20.5 (10.7, 61.0) months. A total of 25 patients (59.5%) were diagnosed with Infantile epileptic spasm syndrome (IESS), 4 patients (9.5%) with Lennox–Gastaut syndrome (LGS). The median time to the first seizure was 9 (2.0, 32.0) months. The median time to the diagnosis of epilepsy before KD initiation was 7.5 (7.0, 9.0) months. Patients initiated KD with a median of 3ASMs. Based on cranial MRI, the patients were divided into an acquired brain injury group (27 cases, 64.3%) and MCD group (15 cases, 35.7%). In the acquired brain injury group (Group 1), there were nine cases of hypoxic–ischemic encephalopathy (33.3%), four cases of neonatal hypoglycemic brain injury (14.8%), four cases of intracranial hemorrhage (14.8%), three cases of sequelae after intracranial infection (11.1%), two cases following intracranial surgery (9.1%), one case of cerebral hemangioma (3.7%), and four other cases (14.8%). In the MCD group (Group 2), there were nine cases of FCD (45%), three cases of megalencephaly (15%), two cases of tuberous sclerosis (10%), one case of gray matter heterotopia (6.7%). The brain MRI findings of several patients are shown in Figure 1. The proportion of diseases is shown in Figure 2. The specific clinical characteristics are detailed in Table 1.

**Table 1:** Clinical profile of patients with drug-resistant structural epilepsy at baseline.

Baseline characteristics	Total (*n* = 42)	Acquired brain injury (*n* = 27)	MCD (*n* = 15)	*P*
Age (median, months)	20.5 (10.7, 61.0)	22.0 (12.0, 61.0)	15.0 (7.0, 50.0)	0.438
Sex (*n*, %)				0.654
male	29 (69.0%)	18 (66.7%)	11 (73.3%)	
female	13 (31.0%)	9 (33.3%)	4 (26.7%)	
Age of seizure onset (mean, months)	9.0 (2.0, 32.0)	10.0 (2.0, 30.0)	6.0 (2.0, 14.0)	0.783
Duration of epilepsy before KD initiation (median, months)	7.5 (7.0, 9.0)	8.0 (7.0, 9.0)	7.0 (6.0, 8.0)	0.521
≥3 ASMs before KD (*n*, %)	22 (52.4%)	13 (48.1%)	9 (60.0%)	0.461
Seizure frequency ≥ 5/ day (*n*, %)	25 (59.5%)	15 (55.6%)	10 (66.7%)	0.482
Developmental delay (*n*, %)	40 (95.2%)	27 (100.0%)	13 (86.7%)	0.122
Epilepsy syndromes (*n*, %)				0.885
IESS	25 (59.5%)	16 (59.3%)	9 (60.0%)	
LGS	4 (9.5%)	3 (11.1%)	1 (6.7%)	
Cannot be classified	13 (31.0%)	8 (29.6%)	5 (33.3%)	
Number of seizure types (*n*, %)				0.611
1	19 (45.2%)	13 (48.1%)	6 (40.0%)	
≥2	23 (54.8%)	14 (51.9%)	9 (60.0%)	
Types of seizures (*n*, %)				0.682
Epileptic spasms	26 (61.9%)	18 (66.7%)	8 (53.3%)	
generalized tonic–clonic seizure	14 (33.3%)	8 (29.6%)	6 (40.0%)	
Others	2 (4.76%)	1 (3.70%)	1 (6.67%)	
EEG (*n*, %)				0.114
Hypsarrhythmia	12 (28.6%)	5 (18.5%)	7 (46.7%)	
Others	30 (71.4%)	22 (81.5%)	8 (53.3%)	

Among the 42 pediatric patients, there were 1 to 4 types of seizure manifestations during the course of illness, with multiple types being the majority. A total of 23 patients (54.8%) experienced multiple types of seizures, while 19 patients (45.2%) had only one type. Motor symptoms were most commonly observed in the seizure semiology. During the course of illness, 26 patients (61.9%) exhibited epileptic spasms, 12 (28.6%) had tonic seizures, 14 (33.3%) experienced generalized tonic–clonic seizures, 6 (14.3%) had myoclonic seizures, 1 (2.4%) exhibited clonic seizures, 2 (4.8%) had atonic seizures, 2 (4.8%) had absence seizures, and 1 patient (2.4%) had status epilepticus. Among the 42 pediatric patients, Idiopathic Epilepsy with Specific Syndromes (IESS) was the most common, accounting for 25 patients (59.5%), followed by Lennox–Gastaut Syndrome (LGS) in four patients (9.5%). The remaining patients could not be classified into specific epilepsy syndromes. The specific clinical characteristics are detailed in Table 1.

### 3.2 Response to KD

**Figure 3:**
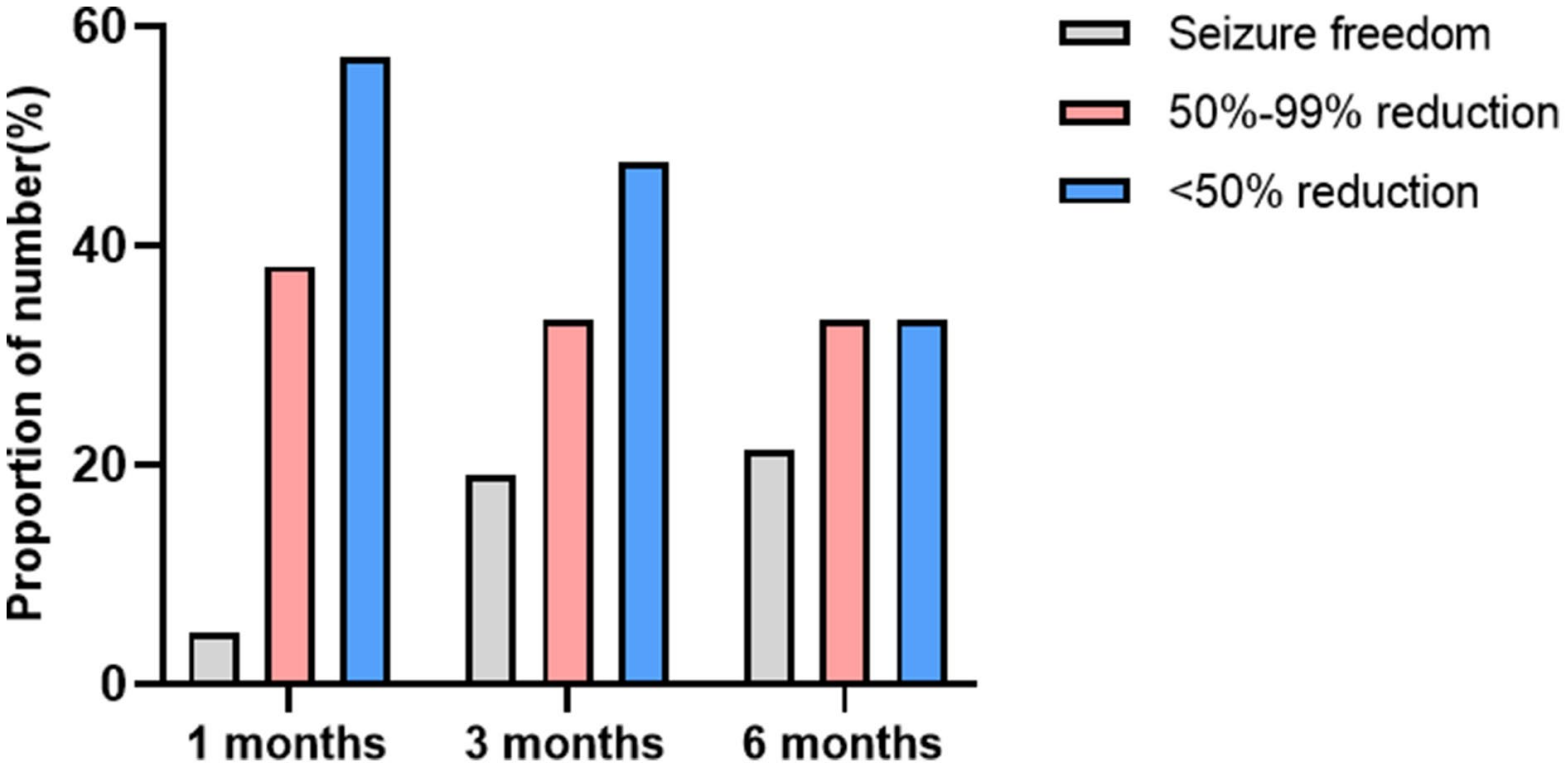
The proportion of number responses to the KD treatment at 1, 3, and 6 months.

**Figure 4:**
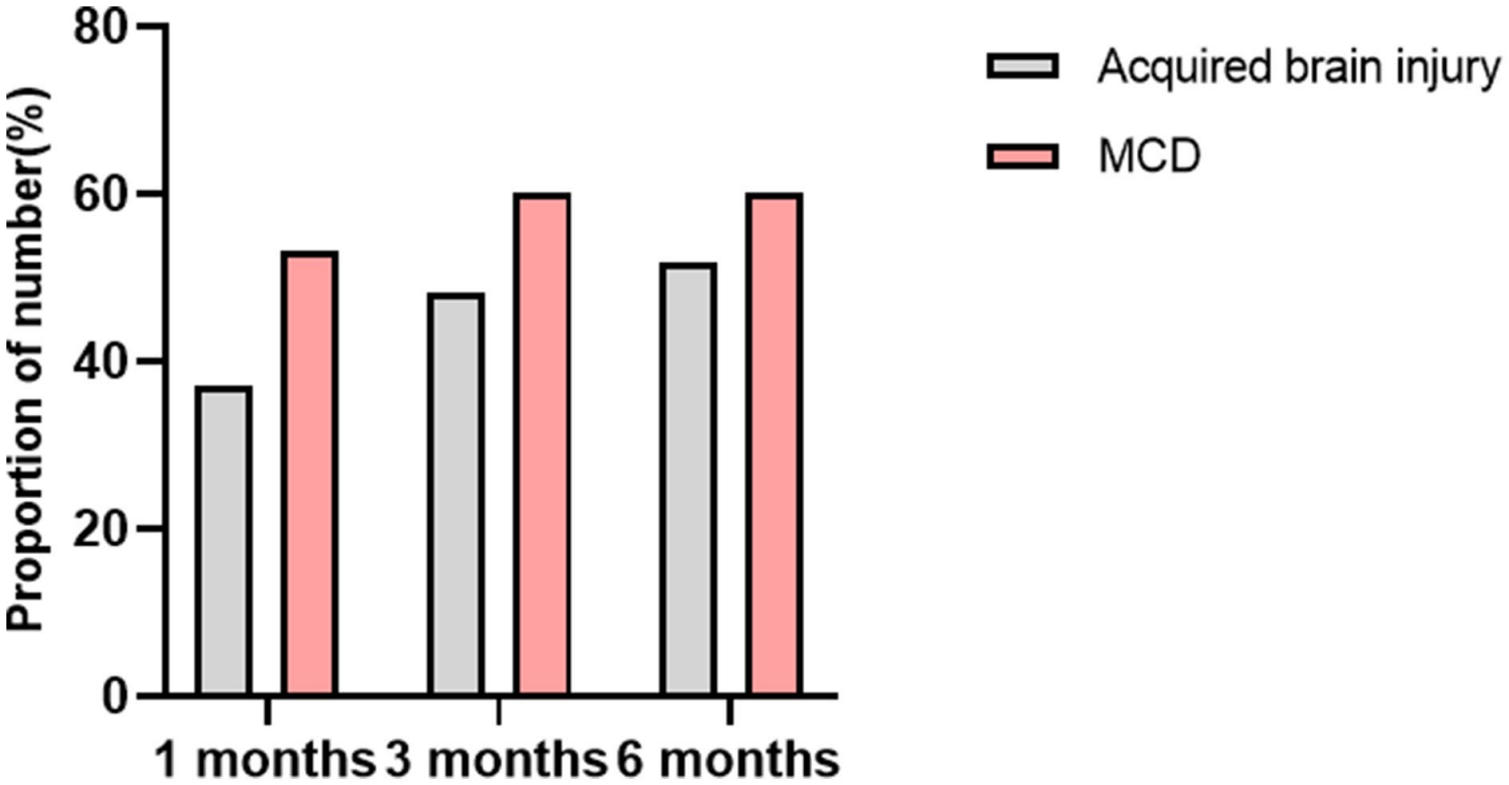
The proportion of effective control over seizures in cases of MCD and secondary brain injuries after 1, 3, and 6 months of KD treatment.

The average duration of KD treatment for the children was 7.50 (7.0, 9.0) months. At 1 month, 3 months, and 6 months of follow-up, the seizure-free rates were 4.8% (2/42), 19.0% (8/42), and 21.4% (9/42), respectively. The effective seizure control rates were 42.9% (18/42), 52.4% (22/42), and 54.8% (23/42) at these respective time points. When compared to the acquired brain injury group, the MCD group showed a higher rate of effective seizure control: 53.3% (8/15), 60.0% (9/15), and 60.0% (9/15). The seizure-free rates were 6.7% (1/15), 26.7% (4/15), and 26.7% (4/15). A summary of the response to KD treatment at 1 month, 3 months, and 6 months different follow-up time stages is shown in Figure 3. A summary of the response to KD treatment of proportion of effective control over seizures in cases of MCD and secondary brain injuries after 1, 3, and 6 months of KD treatment in Figure 4. At the 3-month follow-up, 69% (29/42) of the children showed improvements in cognitive and behavioral aspects, including motor skills, language, emotions, and facial expressions. At the 3-month follow-up, there was no significant correlation between blood ketone levels and seizure control (*p* > 0.05).

At the 3-month follow-up, among the acquired brain injury group, 12 cases (12/27, 48.1%) achieved effective seizure control, with four cases (4/27, 14.8%) being seizure-free. In the MCD group, nine cases (9/15, 60.0%) showed effective seizure control, with four cases (4/15, 26.7%) experiencing no seizures. Statistical analysis using Pearson chi-square test suggested no significant difference between the two groups. However, the trend in normal distribution indicated that children in the MCD group responded better to KD treatment than those in the acquired brain injury group.

Among the 20 children in the MCD group, those with FCD showed the highest reduction in seizures, responding most favorably to KD treatment. At the 3-month follow-up, 6 cases (6/9, 66.7%) with FCD achieved effective seizure control, A total of two cases (2/2, 100%) children with tuberous sclerosis achieved effective seizure control after 3 months of ketogenic diet. In the acquired brain injury group, two cases (2/4, 50.0%) those with sequelae of Hypoglycemic encephalopathy showed the best seizure control, while3 cases (3/9, 33.3%) children with HIE exhibited a poorer response. The reduction rate in seizure control for these structural causes was low.

### 3.3 Retention rate and reasons for discontinuation of KD

Treatment at 3 months of KD treatment, 0 children withdrew, resulting in a retention rate of 100%. At 6 months, five children withdrew, yielding a retention rate of 88.1%. Among the five children who discontinued KD treatment, one case had poor efficacy, four cases had poor compliance.

### 3.4 Adverse reactions analysis

Among the 42 children, 13 experienced varying degrees of adverse reactions, accounting for 31.0%. Among these, 77% (10/13 cases) reported mild symptoms such as diarrhea, constipation, vomiting, and poor appetite. One child terminated KD treatment due to eating refusal. Additionally, one child developed a rash, another had bladder sediment, and one presented with gallstones, but these conditions resolved upon subsequent examinations and follow-ups.

### 3.5 Possible factors regarding the likelihood of seizure-free response to the diet therapy at 3 months

The study also compared the 34 patients with ongoing seizure 3 months after initiation of the diet therapy to the eight patients who experienced seizure-free with the commencement of the dietary treatment. A higher chance of a seizure-free response to the dietary treatment was predicted by responsiveness to the diet at 1 month, number of seizure types and seizure frequency ≥ 5/day (*p* = 0.007, *p* = 0.01, and *p* = 0.007, respectively). The specific factors are detailed in Table 2.

**Table 2:** Possible factors regarding the likelihood of seizure-free response to the diet therapy at 3 months.

Factors	Seizure-free (*n* = 8)	Not seizure-free (*n* = 34)	*P*
Age (*n*, %)			0.294
≤ 2 years	3 (37.5%)	19 (55.9%)	
>2 years	5 (62.5%)	15 (44.1%)	
Sex (*n*, %)			0.522
Female	2 (25.0%)	11 (32.3%)	
Male	6 (75.0%)	23 (67.6%)	
The number of response to KD at 1 month (*n*, %)	7 (87.5%)	11 (32.4%)	0.007
≥50% reduction	7 (87.5%)	11 (32.4%)	
<50% reduction	1 (12.5%)	23 (67.6%)	
Age of seizure onset (median, months)	8.0 (2.0, 52.0)	9.0 (0.03, 125.0)	0.373
Duration of seizure onset to KD Initiation (median, months)	12.0 (1.0, 55.0)	6.0 (1.0, 72.0)	0.323
Number of ≥3ASMs before KD initiation (*n*, %)	6 (75.0%)	16 (47.1%)	0.152
Seizure frequency (*n*, %)			0.007
≥5/day	1 (12.5%)	23 (67.6%)	
<5/day	7 (87.5%)	11 (32.4%)	
Developmental delay	8 (100.0%)	32 (94.1%)	0.652
Structural etiology (*n*, %)			0.294
Acquired brain injury	3 (37.5%)	19 (55.9%)	
MCD	5 (62.5%)	15 (44.1%)	
Number of seizure types (*n*, %)			0.010
=1	7 (87.5%)	12 (35.3%)	
≥2	1 (12.5%)	22 (64.7%)	
Types of seizures (*n*, %)			0.355
Epileptic spasms	4 (50.0%)	12 (35.3%)	
Other	4 (50.0%)	22 (64.7%)	
Hypsarrhythmia in EEG (*n*, %)	1 (12.5%)	10 (29.4%)	0.312

## 4 Discussion

KD is a high-fat, low-carbohydrate, and adequate protein dietary regimen. Its specific mechanisms of action remain unclear but may result from various factors, primarily by simulating a “fasting state.” Making ketone bodies in fat metabolism replace glucose as the main source of energy for the central nervous system, inhibiting or blocking glutamate synaptic transmission, voltage-gated sodium and calcium channels, the mammalian target of rapamycin (mTOR) signaling pathway, activating ATP-sensitive potassium channels, reducing oxidative stress, promoting gamma-aminobutyric acid (GABA) synthesis, enhancing mitochondrial function, and increasing adenosine levels. These mechanisms collectively exert antiseizure, anti-inflammatory, and neuroprotective effects, aiming to treat resistant epilepsy (10, 11).

Epilepsy is a common neurological disorder in children, affecting approximately one-third of the affected children who, despite standardized antiepileptic treatment, continue to experience uncontrolled seizures, progressing to DRE. Seizures caused by structural etiologies are often challenging to control effectively with conventional antiepileptic drugs. Structural causes represent the most prevalent factors underlying seizures (7), with MCD being predominant among congenital anomalies and hypoxic–ischemic brain injury being more common in acquired factors (5, 6, 12). A cross-sectional, multicenter study highlighted perinatal injury as the most common structural cause of epilepsy (12), and perinatal trauma remains a frequent cause in infants under 2 years of age (13, 14). Neuroimaging revealing structural alterations suggests a higher likelihood of drug resistance, often associated with poor early medication efficacy. Different lesion characteristics and locations may indicate varying rates of drug resistance (15). Numerous studies consistently indicate that structural causes are common reasons to the development of resistant epilepsy in newly diagnosed pediatric patients (15, 16).

A cross-sectional study examined the impact of the KD on reducing resistant seizures in Saudi Arabian children, demonstrating an improvement in seizures associated with resistant epilepsy in children (17). Marisa et al.’s (18) retrospective study provided evidence that the ketogenic diet effectively controls seizures in epileptic encephalopathies (18). Research has demonstrated the efficacy of the ketogenic diet in various drug-resistant epilepsy cases in children (19). However, limited literature addresses the specific impact of the ketogenic diet on resistant epilepsy resulting from structural causes. In this retrospective study, we investigated 42 cases of drug-resistant epilepsy due to structural causes treated with KD, analyzing its efficacy and safety. At the 3-month follow-up, the seizure-free rate reached 19% (8/42), with an effective seizure control rate of 52.4% (22/42). Villaluz et al. (20) acquired structural anomalies are examined in this literature and achieved a seizure control rate of 66.6% at the 3-month follow-up (20), while Dou et al. (21) conducted a retrospective study on 23 children with drug-resistant epilepsy resulting from structural causes, reporting a seizure control rate of 60.9% at 3 months (21). Therefore, relevant research results have proved that ketogenic diet is effective for the treatment of children with drug-resistant epilepsy caused by structural causes, which provides a basis for clinicians to choose ketogenic diet as a treatment plan.

In this study, the efficacy of seizure control in the MCD group was found to be higher, as compared to the acquired brain injury group, contrary to previous research findings (21). Among the 15 children in the MCD group, those with FCD demonstrated the highest reduction in seizure frequency and the most favorable response to KD treatment. At the 3-month follow-up, 66.7% (6/9) of the FCD patients achieved effective seizure control. A long-term study investigating the therapeutic outcomes of KD in drug-resistant epilepsy resulting from FCD reported that, at 3 months, 61.7% of patients achieved effective seizure control, with 44.7% experiencing complete seizure freedom (22). The exact reasons for this phenomenon remain unclear, but it may be associated with the immature development of the cerebral cortex, leading to a preference for ketone bodies over glucose as an energy substrate. The immature cerebral cortex may better utilize ketone bodies (23). Surgical intervention can provide complete seizure control for FCD patients with identified lesions, but due to the invasive nature of surgery, it may cause serious functional impairment diseases, so the surgical treatment is still a serious challenge, therefore, the ketogenic diet offers a treatment alternative for children with FCD-associated epilepsy. The results showed that seizures were effectively controlled at 3 months of age in 2 children with tuberous sclerosis. Tuberous sclerosis is mainly caused by TSC1 or TSC2 gene mutation, which leads to a series of clinical manifestations such as enhanced mTOR pathway activity and seizures. Animal experimental studies have shown that ketogenic diet may reduce seizures in children with tuberous sclerosis by inhibiting mTOR pathway signaling and blocking mTOR pathway activation (24, 25). Additionally, in the hemimegaloencephaly group, one out of three cases demonstrated effective seizure control at the 3-month follow-up. However, the subsequent treatment response could not be determined as the patients voluntarily discontinued the ketogenic diet after 6 months.

In the acquired brain injury group, the subgroup with neonatal hypoglycemic brain injury exhibited the most favorable outcomes in terms of seizure control. The effective seizure control rate for epilepsy with structural etiology due to sequelae from neonatal hypoglycemic brain injury was highest, reaching 50% (2/4 cases) at the 3-month follow-up. Conversely, structural causes such as HIE demonstrated a lower response to KD, with a reduced rate of seizure control. Notably, a study by Dou et al. (21) found that epilepsy patients with a history of HIE had the highest seizure reduction rate (100%), whereas those with a history of hypoglycemic encephalopathy had a less favorable response to KD (21). In this study, the response rate to the ketogenic diet for drug-resistant epilepsy resulting from sequelae of intracranial infections in the acquired brain injury group was 33.3% (1/3 cases), possibly linked to the anti-inflammatory effects of the ketogenic diet (26). The exact mechanism is still unknown and needs to be confirmed by the inclusion of more samples.

Furthermore, 69% (29/42) of the children in this study showed improvements in cognitive and behavioral aspects, including motor skills, language, emotions, and facial expressions. In clinical practice, behavioral changes, cognitive improvements, and enhancements in motor and language skills are vital indicators of recovery from brain injuries. Our findings align with previous research, suggesting that the ketogenic diet can enhance cognitive and behavioral abilities in children with epilepsy (21, 27). An animal experiment indicated that rats with brain injuries exhibited superior cognitive abilities when subjected to a ketogenic diet compared to the control group (28). Qiao et al. (29) found through animal experiments that ketogenic diet can further improve cognitive function by regulating ER stress and improving synaptic structural and functional plasticity. However, the underlying molecular mechanisms of protective cognitive function need to be further studied.

The blood ketone levels at the 3-month follow-up did not show a significant correlation with seizure control. This is consistent with the results of previous studies (21). This lack of correlation may be attributed to the limited sample size in our study. Due to differences in dietary habits in China, achieving ideal blood *β*-hydroxybutyrate (BOH) concentrations may be challenging for children accustomed to carbohydrate-rich diets, and maintaining these levels could also be difficult.

The study also compared the 34 patients with ongoing seizure 3 months after initiation of the diet therapy to the eight patients who experienced seizure-free with the commencement of the dietary treatment. We found a higher chance of a seizure-free response to the dietary treatment was predicted by responsiveness to the diet at 1 month, number of seizure types and seizure frequency ≥ 5/day (*p* = 0.007, *p* = 0.01, and *p* = 0.007, respectively). We wondered whether it could be used as a potential predictor of seizure control. However, a large number of samples are needed for further confirmation.

Analysis of the reasons for children discontinuing the ketogenic diet revealed a significant proportion attributed to poor adherence. This is largely due to the fact that the ketogenic diet deviates from traditional Chinese dietary habits and involves a complex and cumbersome process. Caregivers are required to calculate the content of carbohydrates, proteins, and fats in each meal, measure blood glucose and ketone levels, and keep a detailed ketogenic diary. The entire process places a considerable test on the patience and persistence of the parents. Currently, specialized nutritionists tailor individualized diets for children based on their tolerance, solve all kinds of problems encountered by parents of children in time, guide the adjustment of recipes, and under the joint management of doctors, nutritionists, and parents, adherence to the ketogenic diet can be improved.

The adverse reactions associated with the ketogenic diet primarily involve gastrointestinal issues, with 77.0% (10/13 cases) experiencing symptoms such as diarrhea, constipation, vomiting, and poor appetite. Most of these symptoms were mild, and only one patient discontinued KD treatment due to poor appetite. Additionally, one patient developed a rash, one had bladder sediment, and another developed gallstone, all of which resolved upon follow-up examinations, consistent with findings from previous studies (30–32).

Include a small sample size, preventing meaningful comparisons in some groups. Further studies with larger sample sizes are needed to confirm these results. Prospective studies can also be conducted in the future. Focusing on cognitive and behavioral improvements in children, using quantitative scoring methods, could address the subjective nature of the evaluations. Longer-term follow-up studies are needed in which behavioural and cognitive functions in children are examined more comprehensively. The ketogenic diet appears to be effective in reducing seizures in Chinese children with drug-resistant epilepsy resulting from structural etiology. The group with MCD exhibits higher sensitivity to the ketogenic diet, especially in cases of FCD where seizure control is most effective. Among the acquired brain injury group, the subgroup with sequelae from neonatal hypoglycemic brain injury demonstrated the highest efficacy in seizure control. Since this is a small study group, it is difficult to attribute these results to all Chinese children. Future studies with larger sample sizes are warranted to confirm these findings and analyze influencing factors.

- KD is a safe and effective treatment for drug resistant epilepsy caused by structural etiology, with the added benefit of improving behavioral and cognitive abilities in children. Individualized plans are formulated by dietitian, maintaining blood ketone levels between 3 and 5 mmol/L, blood glucose between 4 and 5 mmol/L, and urine ketones between 3+ and 4+. Early intervention with KD is recommended for this population. At the 3-month follow-up, 69% (29/42) of the children showed improvements in cognitive and behavioral aspects, including motor skills, language, emotions, and facial expressions.
- The children were categorized into acquired brain injury group and MCD group based on the etiology. The trend in normal distribution indicated that children in the MCD group responded better to KD treatment than those in the acquired brain injury group. Among the 15 children in the MCD group, those with FCD showed the highest reduction in seizures, responding most favorably to KD treatment. In the acquired brain injury group, those with sequelae of Hypoglycemic encephalopathy showed the best seizure control, while children with HIE exhibited a poorer response.
- The study also compared the 34 patients with ongoing seizure 3 months after initiation of the diet therapy to the eight patients who experienced seizure-free with the commencement of the dietary treatment. We found that a higher likelihood of achieving a seizure-free response to KD was associated with a positive response to the diet after just 1 month, a higher number of seizure types, and a seizure frequency of more than 5 per day. We wondered it could be used as a potential predictor of seizure control.

## Data Availability

The raw data supporting the conclusions of this article will be made available by the authors, without undue reservation.
